# Crossroads between membrane trafficking machinery and copper homeostasis in the nerve system

**DOI:** 10.1098/rsob.210128

**Published:** 2021-12-01

**Authors:** Meng-Hsuan Wen, Xihong Xie, Pei-San Huang, Karen Yang, Tai-Yen Chen

**Affiliations:** ^1^ Department of Chemistry, University of Houston, Houston, TX 77204, USA; ^2^ Department of Molecular Biology, Princeton University, Princeton, NJ 08544, USA

**Keywords:** copper transporters, membrane trafficking regulators, neurodegenerative diseases, CTR1, ATP7A/B

## Abstract

Imbalanced copper homeostasis and perturbation of membrane trafficking are two common symptoms that have been associated with the pathogenesis of neurodegenerative and neurodevelopmental diseases. Accumulating evidence from biophysical, cellular and *in vivo* studies suggest that membrane trafficking orchestrates both copper homeostasis and neural functions—however, a systematic review of how copper homeostasis and membrane trafficking interplays in neurons remains lacking. Here, we summarize current knowledge of the general trafficking itineraries for copper transporters and highlight several critical membrane trafficking regulators in maintaining copper homeostasis. We discuss how membrane trafficking regulators may alter copper transporter distribution in different membrane compartments to regulate intracellular copper homeostasis. Using Parkinson's disease and MEDNIK as examples, we further elaborate how misregulated trafficking regulators may interplay parallelly or synergistically with copper dyshomeostasis in devastating pathogenesis in neurodegenerative diseases. Finally, we explore multiple unsolved questions and highlight the existing challenges to understand how copper homeostasis is modulated through membrane trafficking.

## Graphical abstract

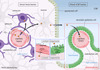

## 1. Introduction

Imbalanced copper (Cu) homeostasis has been associated with the pathogenesis of neurodegenerative disorders such as Alzheimer's disease (AD), Parkinson's disease (PD) and familial amyotrophic lateral sclerosis (ALS) [1–3]. Multiple studies have suggested that Cu influences the regulation and aggregation of the AD hallmark protein, amyloid-β and tau, as well as interacts with α-synuclein to produce toxic oligomers in PD. On the other hand, perturbation of membrane trafficking, such as lysosomal failure or impaired endocytic recycling, has also emerged as a common symptom in many neurodegenerative diseases [4,5]. The concurrent observation of perturbations in both pathways in diseases raises an interesting question: how can membrane trafficking machinery and Cu homeostasis interplay in the nerve system?

From the circulation perspective, the blood–brain barrier (BBB) is the primary route for Cu uptake into the brain's central nervous system (CNS). Cu is associated with soluble Cu-carriers and transported across membrane compartments of endothelial cells to prevent oxidative damage from free Cu ions. The uptake, efflux and distribution of Cu across cell membranes are mediated by membrane-integrated Cu transporters, including Cu transporter 1 (CTR1), divalent metal transporters (DMTs) and P-type ATPases, ATP7A and ATP7B, respectively. These transporters further distribute Cu to different organelles via their corresponding chaperones in a Cu-dependent manner.

Since these Cu transporters reside in different cellular compartments, the trafficking between membrane compartments serves as the regulatory mechanism to balance Cu uptake and Cu excretion [6–8]. Many cellular proteins, including coat proteins, adaptor and effector proteins, and cytoskeletons, coordinate with each other, forming complex and sophisticated regulatory machineries that determine the travelling fates of intracellular membrane compartments carrying Cu transporters. Dysregulation of membrane trafficking could result in misplacement of Cu transporters, imbalance between Cu uptake and exclusion and immaturity of functional cuproproteins, thus perturbing Cu distribution [7–10]. These findings highlight the importance of membrane trafficking in modulating Cu homeostasis. It is well known that neural cells are the most polarized cells and have the heaviest membrane trafficking activity, contributing to the high demand for long-range transport and neuronal excitability [11–13]. It may not be surprising to see a close relationship between Cu homeostasis and membrane trafficking in neural cells.

This review aims to provide a general view of the current understanding of Cu distribution in mammalian brains. We outline the importance of membrane trafficking machinery in maintaining proper Cu distribution and cellular Cu homeostasis, with particular attention to the key regulators responsible for distributing and recycling Cu transports across different membrane compartments in cells. We also use diseases showing both neuropathy and Cu-dysregulation symptoms as examples to delineate how membrane trafficking and Cu homeostasis may interplay to further exacerbate the symptoms in the nerve system. Considering the majority of current understanding about the trafficking regulation of Cu transporters is done in the non-neuronal system, we explore multiple unsolved questions and highlight the existing challenges. The molecular picture of interplaying pathways between membrane trafficking machinery and Cu homeostasis could help understand Cu transporters' physiological configurations, signalling and behaviour dynamics in maintaining neuronal Cu balance.

## 2. Copper transport regulation in the brain

As a catalytic and structural cofactor, Cu constitutes the active sites of many metalloproteins to enable electron transfer, removal of reactive oxygen species (ROS), production of neurotransmitters and neuronal differentiation [14–17]. Although essential, Cu's redox properties also make it detrimental when dysregulated [18,19]. The BBB and the blood–cerebrospinal fluid (CSF) barrier (BCB) are the essential gatekeeping structures for Cu's entrance or exit from the brain. After passing the BBB, Cu eventually enters the neurons. Neurons require timely adjustment of intracellular redox status and distribution of Cu for proper neuron-chemical activities and general metabolism [16,17,20]. Following the entry into neurons via plasma membrane (PM) transporters [21–23], Cu^+^ is transferred by chaperones to distinct intracellular compartments for redox activity, protein maturation and Cu efflux, or sequestered to metallothionein and abundant Cu-binding tri-peptide glutathione for Cu storage. Here, we summarize the working principles of Cu-binding proteins in the uptake, distribution and storage and secretory pathways, as well as their connections to the neuronal functions.

### 2.1. Cu circulation in and out of the brain: BBB and BCB

Next to the liver, the brain is the second most Cu-abundant organ in the body (approx. 5 µg g^−1^ wet tissue weight). The Cu concentration in CSF (approx. 0.02 µg ml^−1^) is much lower than in blood (approx. 0.9 µg ml^−1^), indicating Cu circulation to the brain is strictly regulated [24–26]. Humans acquire Cu from their diet and re-distribute it to different organs through the circulation except for the brain. The microenvironment of the brain is separated from peripheral circulation by the BBB and the BCB [27]. Studies suggest BBB and BCB are the primary routes for Cu to enter and export to and from the brain, respectively, to maintain Cu homeostasis in CNS from the circulation [28]. The form of Cu transferring across the BBB is still unclear. In blood, Cu is mainly bound to ceruloplasmin, albumin, transcuprein and amino acids [29]. Ceruloplasmin-bound and albumin-bound Cu are likely not the forms transporting through the brain since their transgenic null models show no significant difference in brain Cu content compared to the wild-types [27,30,31]. Even though it has been demonstrated that free Cu transport into the brain is much faster than ceruloplasmin or albumin-bound Cu [27], there is still no consensus if free Cu is the species that enter the BBB, especially considering most Cu-binding proteins have pico-molar Cu-binding affinity.

**Figure 1.**
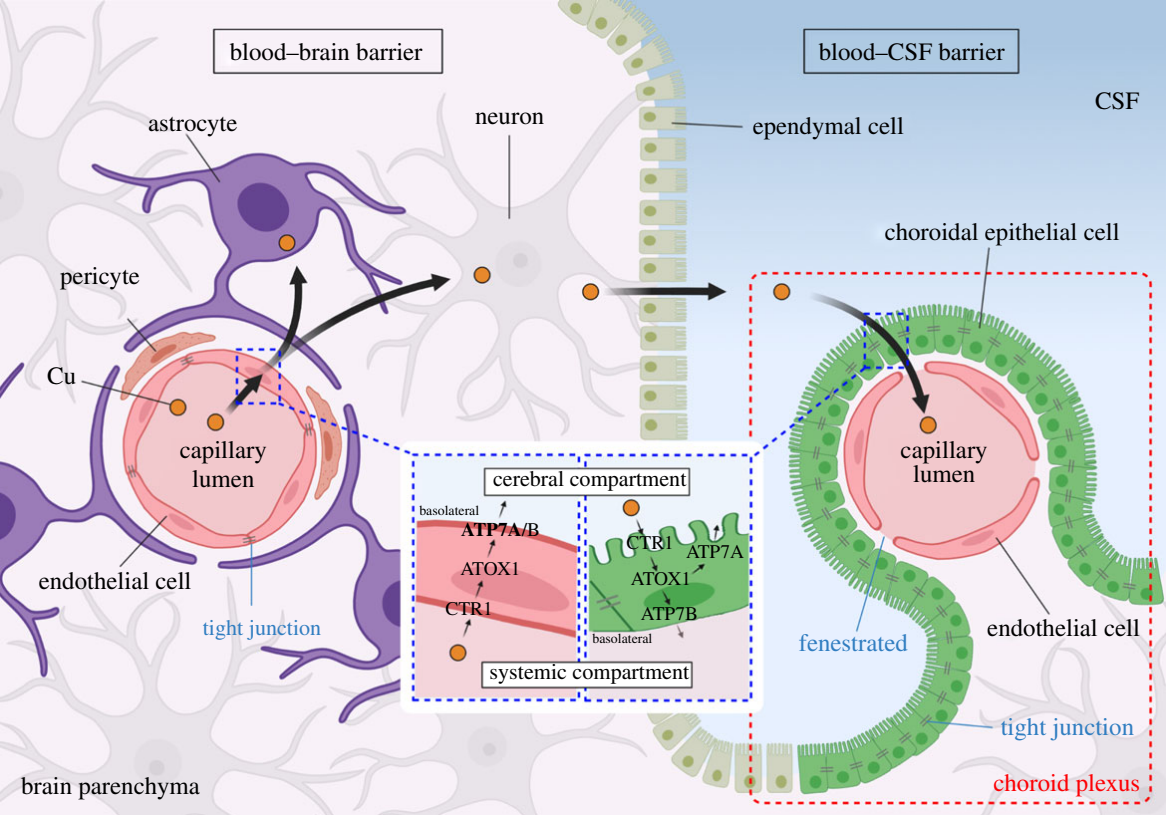
Copper transportation across brain barriers BBB and BCB. Cu is uptake from the systemic circulation via BBB endothelial cell CTR1 and released to the parenchyma by ATP7A/B. Excess Cu flow to the CSF where it can be removed by CTR1 in BCB choroidal epithelial cells and released back into the blood by ATP7A/B.

The BBB comprises the endothelial cells of the cerebral capillary, pericytes embedded in the capillary basement membrane and perivascular feet of astrocytes (figure 1). This unique structure makes the BBB a highly selective semipermeable barrier that controls Cu transport from blood circulation to the interstitial fluid of the brain and distributes throughout the brain. By contrast, the BCB separates blood from the CSF produced in the choroid plexuses of the ventricles of the brain. The structural basis of the BCB includes the tight junctions between choroidal epithelial cells, the capillary basement membrane and endothelium cells containing fenestrations. To enter the brain, Cu needs to pass across these barriers through Cu transporter proteins. High-affinity CTR1, antioxidant 1 (ATOX1) and P-type ATP7ases copper-transporting alpha/beta (ATP7A/B) mediate Cu trafficking within the BBB and BCB. The postulated model starts with Cu uptake from the blood by CTR1 in capillary endothelial cells. Once Cu is obtained from Cu chaperones, ATP7A translocates to the abluminal membrane and releases Cu into brain interstitial fluid for neuronal activities. Under Cu excess conditions, Cu may flow to the CSF where excessive Cu can be removed by CTR1 in the BCB and released back into the bloodstream by ATP7B. Both ATP7A and ATP7B are present in brain capillary endothelial cells and choroidal epithelial cells but have different relative mRNA levels. Compared to ATP7B, the mRNA expression of ATP7A is 13-times higher in the BBB but four-times lower in the BCB, suggesting that ATP7A plays a major role in transporting Cu from the blood to the brain [32]. For choroidal epithelial cells of the BCB, ATP7A appears to locate toward the apical microvilli while ATP7B toward the basolateral membrane under exposure to excessive Cu [32]. These results are opposite to the observations in placental and intestinal epithelial cells [33,34]. However, the mechanisms involved in the differential allocation of ATP7A and ATP7B in the brain and the discrepancy of dispatch direction between the choroidal epithelia and epithelium outside the brain region are still unclear.

### 2.2. Cellular Cu homeostasis

#### 2.2.1. Cu uptake pathways

**Figure 2.**
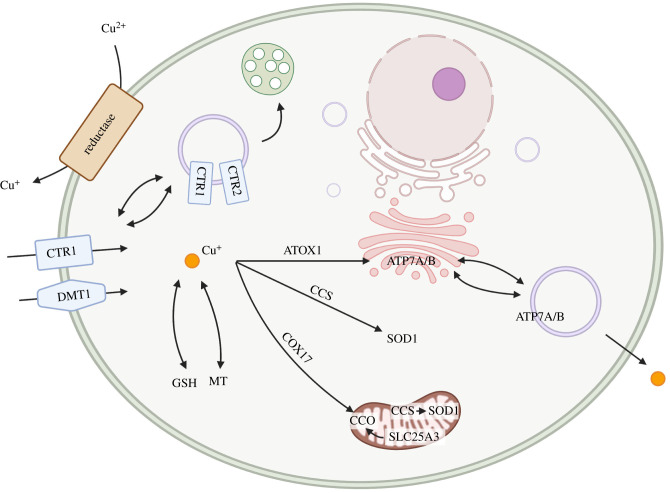
Cellular Cu homeostasis. Cu is reduced by an unknown reductase and taken up into cells by CTR1 and possibly DMT1. Upon entry, Cu is routed to the target destination through the specific protein–protein interactions between Cu chaperones and target proteins: ATOX1 delivers Cu to APT7A and ATP7B for Cu supply in the secretory pathway; CCS distributes Cu to SOD1 for its maturation in both cytosol and mitochondria; COX17 transfers Cu with other subunits to ensemble CCO in mitochondria; SLC25A3 located in the inner membrane of mitochondria is also associated with Cu transport of CCO. Cu can be stored in GSH and MT, while APT7A/B can excrete excess Cu through vesicle trafficking.

CTR1 is responsible for about 70% Cu uptake in human cells [35]. Biochemical analysis and electron microscopic crystallography have revealed that CTR1 subunits assemble into a multimeric complex [36–38]. CTR1 forms a functional trimeric channel in the Cu uptake pathway with a low micromolar affinity (i.e. 0.1–13 µM depending on the tissue type) [21]. Ag^+^ inhibited and reducing agent ascorbate enhanced Cu uptake suggests that CTR1 transports Cu^+^ species. Cu^+^ import into cells is mediated in an energy-independent manner and enhanced by the extracellular acidic environment (low pH) and high K^+^/Na^+^ concentrations [39,40]. Cu uptake is regulated through Cu-dependent vesicular trafficking. At elevated Cu levels, CTR1 undergoes endocytosis from the PM to early endosomes and returns to the PM when normal Cu levels are restored [41–43]. At the molecular level, the relocation of CTR1 is also mediated by Cu transporter 2 (CTR2) [38]. CTR2 is structurally similar to CTR1 and localizes at intracellular vesicular compartments such as endosomes and lysosomes (figure 2). CTR2 stimulates cleavage of the ectodomain of CTR1, implying that it may play a regulatory role in the Cu-dependent mobilization of CTR1 [38].

Ctr1-deficient cells from transgenic mice show about 30% residual Cu transport, suggesting other Cu acquisition pathways. The divalent metal transporters 1 (DMT1), also known as the ferrous iron (Fe^2+^) transporter, transports other metal ions such as manganese, cadmium and Cu across the PM. The partial knockdown of DMT1 resulted in reduced Cu transport and intracellular Cu level in Caco2 cells. Competition studies between iron and Cu uptake indicate DMT1 also selectively transports Cu^+^ [44]. The study from the rat's brain showed that loss of Dmt1 function significantly decreased iron levels but interestingly promoted Cu accumulation in the striatum and hippocampus and upregulated Ctr1 and Atp7A in the hippocampus. This observation implies that altered iron metabolism affects brain Cu transport, even though the molecular mechanism is still largely unknown [45]. Similar crosstalk between Cu and Zn were also observed. Zinc strongly inhibits CTR1-independent Cu transport, suggesting the possibility of the Zrt/IRT-like protein (ZIP) family members involved in Cu uptake, but no direct evidence has been reported [46].

#### 2.2.2. Cu trafficking and storage

Cytosolic Cu is routed to the target destination through specific protein–protein interactions between Cu chaperones and target proteins. ATOX1 is responsible for transporting Cu to ATP7A and ATP7B that supply Cu to the secretory pathway [8]. It interacts and exchanges Cu with the N-terminal Cu-binding domain of ATP7A/B and can transfer up to six Cu ions [47,48]. Furthermore, ATP7A immunofluorescence results between *Atox1*^+/+^ and *Atox1*^−/−^ mouse embryonic fibroblasts (MEFs) suggest that ATOX1 is essential in modulating the Cu-dependent movement of ATP7A from the TGN to the cell surface and determining the threshold for Cu-dependent trafficking of ATP7A [49].

CCS is another chaperone that delivers Cu to and involves the maturation of superoxide dismutase 1 (SOD1). CCS and SOD1 primarily reside in the cytosol but also in the mitochondria and nucleus [50,51]. Excessive Cu downregulates the CCS protein level through a post-translational process, as the mRNA level of CCS does not show any Cu-dependent reduction [52]. The proteasome inhibitor blocking CCS degradation under excessive Cu suggests that Cu regulates CCS expression by modulating its protein degradation [53]. Brady *et al*. identified that CCS is a mediator of Cu delivery to the X-linked inhibitor of apoptosis (XIAP), and XIAP is the E3 ubiquitin ligase of CCS. The study proposes that XIAP-mediated ubiquitination of CCS enhances the ability of CCS to acquire Cu and activate SOD1 under the basal Cu level. Under the elevated Cu level, the Cu-bound CCS transfers Cu to XIAP and is ubiquitinated for proteasomal degradation [54]. Cytochrome *c* oxidase (CCO), a respiratory energy-transducing enzyme, is the main Cu protein complex in mitochondria. COX17, the Cu chaperone for CCO, is implicated in shuttling Cu from cytosolic to mitochondria due to its dual subcellular localization in the cytosol and intermembrane space of mitochondria [55]. It transfers Cu to SCO1, SCO2 and COX11, which is involved in the insertion of Cu into CCO [56–58]. The mammalian phosphate carrier SLC25A3, located in the inner membrane of mitochondria, is also found to transport Cu to CCO [59].

Glutathione (GSH) is a predominant tri-peptide bio-thiol involved in antioxidative defense against ROS and signal transduction [60]. Regarding intracellular Cu homeostasis, Cu–GSH complexes are considered the major exchangeable cytosolic Cu pool and vital in connecting Cu's uptake and cellular trafficking. From the comparison of Cu-binding affinities across major Cu-binding proteins [61] and the fact that GSH transfers Cu to metallothionine [62], ATOX1 [63] and SOD1 [64], GSH has been implicated as the intermediator of the Cu source to other proteins. Chen *et al.* [65] demonstrated that an increased GSH level depletes the exchangeable pool of Cu and upregulates Ctr1 expression in SR3A cells. Maryon *et al*. [66] showed depletion of GSH decreases cellular Cu uptake by CTR1 while depletion of the Cu chaperone ATOX1 and CCS has no effects in HEK293 cells. These studies collectively support GSH as an intermediator of the Cu source and its involvement in Cu uptake. It has been reported that GSH also mediates the export of Cu. GSH regulates the glutathionylation condition of Cu transporters ATP7A and ATP7B [67]. The depletion of GSH affects the vesicular trafficking of ATP7A and leads to Cu accumulation [67].

Metallothioneins (MTs), a family of small (approx. 7 kDa) cysteine-rich proteins that bind zinc and Cu in high stoichiometries (up to 12) [68], are responsible for cellular Cu storage and detoxification [69]. Four MT isoforms, MT-1 to MT-4, were found in mammals. MT-1 and MT-2 exist ubiquitously in the liver, kidney, intestine and brain. MT-3 is mainly located in the brain, and MT-4 in the stratified epithelium [70]. Either Cu overload or Cu deficit has been reported to induce the expression of MTs, indicating MTs must be involved in at least two bio functions [69,71,72]. It is known that the presence of MTs is essential for the survival of cells when ATP7A is deficient [73]. An increased MT level was found in the liver and kidney of Wilson's disease (WD) patients and mouse models [74]. These results collectively suggest the MTs sequester excess Cu to mask Cu toxicity [75]. On the other hand, MTs have been proposed to store Cu to ensure supply for cuproenzymes as MT-null cells show less Cu content and are more sensitive to Cu depletion than wild-type cells [72,76].

#### 2.2.3. Cu-secretory pathways

ATP7A and ATP7B are the essential Cu exporters in balancing intracellular Cu levels. Genetic defects of ATP7A and ATP7B connect to the aetiology of Menkes' diease (MD) and WD, respectively [77,78]. ATP7A/B are responsible for transporting Cu from ATOX1 in the cytoplasm to the Golgi lumen for Cu incorporation into cuproenzymes. The two cuproenzymes that are largely expressed in the nervous system and rely on ATP7A Cu delivery for activation are the peptidyl-α-monooxygenase (PAM) and dopamine-β-hydroxylase (DBH), both belong to the monooxygenase family and require two Cu atoms in each monomer to be functional. Their neuro-specific property provides a connection between Cu homeostasis and the neurological symptoms in diseases with ATP7A/B dysregulation.

PAM is the only enzyme that catalyses the C-terminal α-amidation of neuropeptides and contributes to more than half of all neuropeptides' activities. Its expression is widespread in the CNS, with the highest level in the hypothalamus, hippocampus and neocortex [79]. The relationship between PAM and Cu homeostasis is bidirectional. Transgenic mice with their ATP7A inactivated show decreased levels of several amidated peptides (i.e. decreased PAM function) despite normal PAM protein expression. Thus, compromised PAM functions likely contribute to the neuronal-specific symptoms of patients with ATP7A mutation [80]. On the other hand, PAM is also implied to play a role in Cu metabolism. The transcriptional levels of Atox1 and Cox17 are lower in the pituitary of PAM^+/−^ mice compared to WT mice. While mice lacking PAM do not live past mid-gestation, PAM^+/−^ mice show behaviour and physiological defects that can be mimicked by WT mice under Cu restricted diet. Most of these defects in PAM^+/−^ mice can be reversed using a dietary Cu supplement. However, the peptide amidation level does not show a corresponding increase in these mice, indicating a role for Cu itself in mediating the effects of PAM^+/−^ heterozygosity [19,81,82].

DBH converts dopamine to norepinephrine, a stress hormone and neurotransmitter, in noradrenergic neurons of the locus coeruleus and sympathetic nerve terminals. These neurons send direct and indirect projections throughout the body, including the brain and innervate nearly all the cerebral cortex. It is thus not surprising that dysfunction of DBH links to a wide range of neurological disorders, including neurodegenerative diseases (reviewed in [83]). Current evidence suggests that DBH acquires Cu from ATP7A in the lumen of the *trans*-Golgi network (TGN) [84] and forms tetramers in human cells [85]. Upon functional maturation, there are two pathways for DBH to exit the cells. The majority of DBH is directed to secretory granules, where it catalyses the synthesis of norepinephrine from dopamine. This soluble form of DBH is secreted outside the cell along with norepinephrine in response to neuronal activation. On the other hand, a small population of soluble functional Cu-bound DBH is constantly being secreted out of the cell under resting conditions (without neuronal activation). Although the mechanism of this resting state secretion of DBH is not well understood, Schmidt *et al*. [86] demonstrated that this process is Cu-dependent and differentially regulated by ATP7A and ATP7B. It is unclear whether the cell uses this process to partially handle intracellular Cu balance. Regardless, the study clearly shows that the resting state secretion of DBH is sensitive to Cu balance, indicating Cu homeostasis plays a role in catecholamine metabolism.

ATP7A/B dysregulation affects the activity of cuproenzymes like PAM and DBH, which may account for the neurological symptoms seen in diseases with ATP7A/B mutations. Excessive Cu that is not incorporated into cuproenzymes will be carried by ATP7A/B and expelled from the cell through vesicle trafficking. ATP7A and ATP7B dynamically cycle between TGN, vesicles and PM. Under basal Cu level, ATP7A and ATP7B both reside in TGN to accept Cu from ATOX1 and shuttle it to cuproenzymes in the secretory pathway. It is worth noting that although ATP7A and ATP7B both mediate Cu exclusion, the intracellular destinations (i.e. basolateral and apical membrane) are opposite and vary in different cell types (e.g. choroidal epithelial cells versus hepatocyte) [6,34,87]. In the cerebellum, ATP7A and ATP7B have been proposed to have distinct roles based on their cell-specific distribution, distinct enzymatic characteristics, and only ATP7B colocalized with the Cu-requiring enzyme, ceruloplasmin. With faster transportation, ATP7A is suggested to have a homeostatic role in maintaining intracellular Cu levels. By contrast, ATP7B has a biosynthetic role in mediating the synthesis of Cu-dependent enzymes [88].

### 2.3. Distributions of Cu and Cu transporters in the brain and neurodegenerative disease

**Figure 3.**
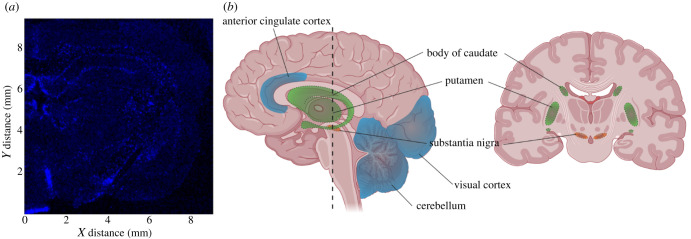
Cu distribution in the brain. (*a*) Fluorescence intensity of Cu normalized to the incident X-ray intensity in mouse brain. Copper is localized to areas surrounding the corpus callosum, the linings of the third ventricle and the choroid plexus. (Figure reprinted with permission from [89]; published by MDPI, 2019.) (*b*) Anatomical regions of human brain for table 1.

Cu is unevenly distributed in the brain (figure 3*a*), and its distribution is altered in AD, PD and ALS patients. In a healthy brain, the grey matter has a higher Cu concentration than white matter, with the highest level in the substantia nigra (figure 3*b* and table 1). For different cell types, histochemical studies with brain slices revealed that glial cells show higher Cu levels than neurons under both physiological and pathological conditions [94].

**Table 1.** Abundance of Cu and Cu transporters.

anatomical region (figure 3*b*)	Cu level^a^	Cu (relative abundance)^b^	CTR1^c^	ATOX1^c^	ATP7A^c^	ATP7B^c^
visual cortex	4.14	0.93	0.65 ± 0.07	1.08 ± 0.21	0.67 ± 0.12	0.58 ± 0.19
anterior cingulate cortex	4.04–57	0.9–1.0	0.68 ± 0.09	1.34 ± 0.25	0.88 ± 0.18	0.47 ± 0.19
body of caudate	5.09–18.46	1.14–1.26	0.74 ± 0.08	1.26 ± 0.20	0.72 ± 0.15	0.84 ± 0.32
putamen	4.47–62	1	0.71 ± 0.12	1.39 ± 0.22	0.70 ± 0.28	0.61 ± 0.20
substantia nigra	11.4–17.42	1.19–2.55	0.73 ± 0.16	2.05 ± 0.60	1.00 ± 0.22	0.33 ± 0.15
cerebellum	4.85–47	0.5–1.08	0.68 ± 0.07	0.92 ± 0.12	2.00 ± 0.45	0.78 ± 0.27

^a^Units are μg g^−1^ wet tissue or dry tissue [90–93].

^b^Normalized to Cu content in the putamen [90–93].

^c^Analysis of western blotting band normalized to β-actin.

Davies *et al.* quantified expression levels of CTR1, ATOX1, ATP7A and ATP7B in the human brain (table 1). CTR1 is ubiquitously expressed in all brain regions except for the Purkinje cells in the cerebellum. There is a similar level among the substantia nigra, anterior cingulate cortex, visual cortex, putamen, the body of caudate and the cerebellum. CTR1 is enriched in the apical surface of ependymal cells in the choroid plexus. In the human visual cortex, anterior cingulate cortex, caudate nucleus and putamen, CTR1 is primarily in the neurons, while in the cerebellum, it is restricted to Bergmann glia [90]. ATP7A and ATP7B protein levels did not show a significant correlation with Cu levels in the brain (table 1). ATP7A protein levels are most prominent in the cerebellum and substantia nigra; ATP7B is dominant in neuronal cells of the hippocampus, glomerular cell layer of the olfactory bulb, granular cell layer of the cerebellum. ATP7A and ATP7B have comparable expression and share a similar cellular distribution in both neuronal soma and proximal fibres in the anterior cingulate cortex. ATP7A and ATP7B are expressed in the striosomes of the caudate nucleus, putamen and cerebellar Purkinje neurons but not in Bergmann glia [90].

Genetic defects on any of these Cu transporters directly cause severe Cu disorder diseases. For example, mutations in ATP7A and ATP7B cause acute defects in early development and are responsible for MD and WD, respectively. In addition, the imbalance of Cu homeostasis in the brain is thought to play an important role in the pathogenesis of many progressive neurodegenerative diseases. Cu levels have been found to be reduced in the substantia nigra and locus coeruleus of the brain from PD patients [90]. In AD, Cu is found in high concentration in Aβ plaques and linked to their deposition [95,96], while decreased Cu levels are found in the hippocampus, amygdala and cerebral cortex [97,98]. ALS patients have elevated Cu levels in the frontal lobe grey matter tissue [99] but reduced intraneuronal Cu levels in the spinal cord [100]. Age-dependent alterations in Cu level are not likely impacted directly by the functional defects of Cu transporters *per se*. Instead, they may be related to the accumulative impacts caused by trafficking dysregulation of Cu transporters CTR1, ATP7A and ATP7B.

## 3. Membrane compartments and their communications involved in regulating intracellular Cu homeostasis

**Figure 4.**
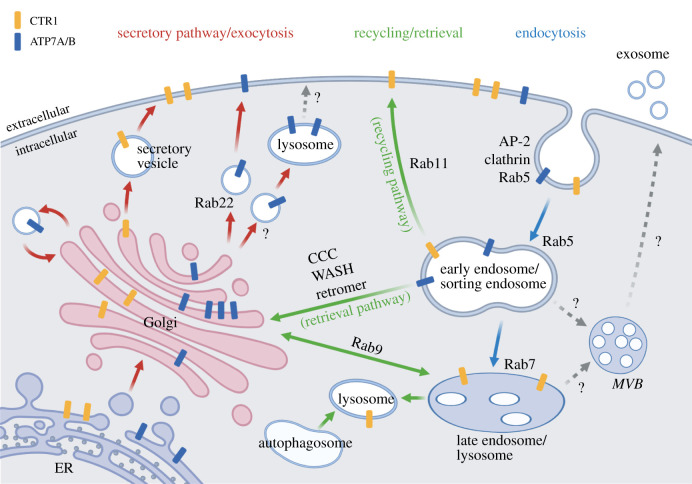
Intracellular membrane trafficking of Cu-related transporters. In a normal Cu environment, newly synthesized CTR1 and ATP7A/B travel along the secretory pathway (red arrows) from the ER, Golgi to PM, which allocates ATP7A/B at the TGN and CTR1 at the PM. In an elevated Cu condition, ATP7A/B further travels to the PM by exocytosis and/or travels to intermediated vesicles which later will be incorporated into early/sorting endosomes. Meanwhile, CTR1 is internalized via endocytosis (blue arrows) and stays at internalized vesicles temporally or travels further to lysosomes for permanent degradation. When the Cu environment is restored to normal, CTR1 at internalized vesicles is re-distributed to the PM via the recycling pathway, and ATP7A/B are retrieved back to the TGN via the retrieval pathway.

Change of subcellular distribution of membrane proteins, including Cu transporters CTR1 and ATP7A/B, is a pivotal mechanism for regulating their functions. In eukaryotes, the relocation of membrane proteins is governed by a sophisticated membrane network communicated through vesicular trafficking. The trafficking network starts from the membrane protein synthesis pathway, which delivers newly synthesized membrane proteins from the endoplasmic reticulum (ER) via the Golgi apparatus to the PM. When the proteins are removed from the cell surface, the trafficking network delivers internalized surface proteins either for endosomal–autophagy–lysosomal degradation or recycling to other membrane compartments. This vesicular membrane trafficking machinery is coordinated by multiple regulators, including coat proteins, adaptor protein complexes, GTPases, vesicle sorting factors, motor proteins/cytoskeletons, etc., which dictate the identities and destinations of vesicles (figure 4). Dysregulation of membrane trafficking has been shown to misplace Cu transporters, which leads to intracellular Cu imbalance. Concomitantly, dysfunctional metal ion homeostasis may result in neurodegeneration and neuroinflammation, contributing to the development of several neurodegenerative diseases [101]. Numerous membrane trafficking regulatory proteins have been identified to be closely associated with neuronal dysfunction when mutations occur [5,102]. Therefore, it is likely that the regulation of membrane trafficking is the key to link Cu homeostasis and maintaining neuronal functions. Although extensive studies have been focused on the role of membrane trafficking in the neuronal system, only a limited number of trafficking regulators are identified as responsible for the Cu-induced trafficking of Cu transporters, CTR1 and ATP7A/B. Most of the mechanisms regulating Cu transporter trafficking were currently identified in non-neuronal cells. However, considering that all types of cells share similar trafficking machinery, the dysfunction of those regulators also impacts the pathogenesis of neurodegenerative diseases. Therefore, we introduce key membrane compartments and their associated regulatory mechanisms in regulating the distribution of Cu transporters below.

### 3.1. Key membrane compartments mediating Cu homeostasis

#### 3.1.1. Endoplasmic reticulum

After translation, newly synthesized membrane and secretory proteins, including CTR1 and ATP7A/B, are moved to the ER for proper folding and assembly. Several molecular chaperone families assist this maturation process in ensuring protein integrity. When errors are detected, the proteins are retained for repair or ER-associated degradation. Protein disulfide isomerase (PDI) is a member of the thioredoxin superfamily of redox proteins. PDI assists the target protein's disulfide bond formation and is thus responsible for thiol-dependent quality control. As a redox enzyme with oxidase and reductase activity, PDI regulates the expression and activity of Nox (NADPH oxidase family) proteins, which dedicate the ROS generation in the ER [103]. PDI's Cu-binding ability and catalytical CXXC containing domain a, which is structurally similar to ATOX1, lead to the assumption that PDI may also act as a Cu chaperone and affect Cu disposition although further investigation is needed [104].

In response to excessive Cu, the ER activates the unfolded protein response to avoid ER stress in hepatocytes. Several WD-causing mutations of ATP7B, including the most frequent H1069Q, while still preserving the ATP7B's Cu-transporting activity, result in the protein's extensive ER retention and increased degradation [105–107]. Suppression of the downstream signalling pathways effectively releases the H1069Q mutant from ER to TGN, recovers Cu-dependent trafficking and reduces intracellular Cu levels [108,109]. Electron microscopy reveals that patients with WD have dilated and disorganized ER in hepatocytes, suggesting that Cu influences ER homeostasis, but little is known about this process [110].

In the brain, the toxic effect of Cu is first buffered by astrocytes, and it has been suggested that astrocytes might rely on the ER stress response to protect them from Cu-inducing ROS [111]. To maintain normal Cu levels in the brain, wild-type ATP7B in choroidal epithelial cells of BCB translocates to the basolateral membrane to excrete excess Cu into the blood. ER retention of ATP7B mutants in WD could result in Cu accumulation in the brain. In noradrenergic neurons, where the ATP7A/ATP7B ratio regulates extracellular active DBH (i.e. Cu-bound DBH), extensive ER retention of ATP7B may cause catecholamine misbalance in neurological WD.

#### 3.1.2. Golgi apparatus

The Golgi apparatus is a central node at the intersection of the exocytic and endocytic routes in intracellular membrane trafficking. It plays a crucial role in sorting newly synthesized and recycled proteins and lipids towards their final destinations. It also serves as a biosynthetic centre for glycoproteins and lipids and an active signalling hub [112]. For normal Cu homeostasis, the Golgi apparatus functions as the organelle for PM-targeted cuproenzymes to acquire Cu and become functional [7,113,114]. The Golgi apparatus, specifically at the TGN, harbours ATP7A and ATP7B, which transfer Cu from the cytosol into the Golgi lumen for incorporation into Cu-dependent enzymes such as lysyl oxidase, ceruloplasmin, PAM and DBH [7]. Increases in Cu concentration stimulate the trafficking of ATP7A/B proteins to the recycling vesicles near the PM, where ATP7A/B efflux Cu to ensure proper intracellular Cu fluxes and avoid potentially toxic Cu accumulation. Mutations in ATP7A/B or the membrane trafficking regulators could affect ATP7A/B's exit from or subsequent retrieval to the Golgi apparatus [115–119]. This disturbed trafficking, in turn, disrupts the homeostatic Cu balance, resulting in Cu deficiency (MD) or Cu overload (WD).

At the molecular level, the Golgi apparatus is the location where the transmembrane and secretory proteins undergo *O*-linked glycosylation, a key process that is linked to protein stability and subcellular allocation. CTR1 is a highly glycosylated membrane protein. Glycosylation impairment significantly compromises protein stability and PM abundance of CTR1. Mutation at the glycosylation site (Thr-27) or expression of CTR1 in *O*-glycosylation deficient cells both resulted in proteolytic cleavage of CTR1 [120,121]. Reciprocally, studies in mouse intestinal epithelia demonstrated that Cu availability alters the glycosylation levels of the glycosylated form of Ctr1 in a dose- and time-dependent manner [122]. Glycosylation also has been associated with the stabilization of ATP7A on PMs [123–125]. It has been demonstrated that ATP7A is highly expressed in hippocampus neurons, specifically in the late Golgi [126]. However, unlike CTR1, where glycosylation plays roles in cellular allocation, the role of glycosylation for ATP7A/B function is still unclear.

In neurons, the Golgi apparatus is essential for developing axons and dendrites and maintaining their highly complex polarized morphology. Early occurrence of Golgi pathology is one of the characteristic symptoms of neurodegeneration [127]. Fragmentation of the Golgi apparatus has been reported in numerous pathological non-infectious conditions, including neurodegenerative disorders [128]. The altered organization/function of the Golgi apparatus may impact the secretory performance of the cell and trigger the Golgi stress response, affecting cell survival [112,129]. An interactome study identified several neurodegenerative related gene products, including subunits of Golgi-localized conserved oligomeric Golgi (COG) complex co-isolated with ATP7A [130]. COG is a multisubunit tethering complex that controls membrane trafficking and ensures Golgi homeostasis by orchestrating retrograde vesicle targeting within the Golgi. Cells lacking the COG complex show increased surface levels of ATP7A and display decreased Cu content [130]. Studies on the *Drosophila* neuromuscular junction further revealed that ATP7-mediated Cu homeostasis perturbation led to alterations of mitochondria distribution in synapses and synaptic activities. The downregulation of COG complex subunits can rescue the altered synaptic phenotypes. These results collectively support the critical role of Golgi and Cu in neurodegeneration and neurodevelopmental disorders [131].

#### 3.1.3. Endo-lysosomal system

Endo-lysosomes are a series of discontinuous membrane networks involving the sorting and delivery of membrane compartments and their protein cargo to/from the PM, TGN and lysosomes. Early endosomes, the first membrane compartments invaginated from the PM, play a crucial role in sorting internalized cargos to different intracellular destinations. On entering the endosomal system, the internalized protein cargo is either delivered to the late endosomes/lysosomes for degradation or sorted to recycling endosomes for recycling back to the PM or retrieving back to the TGN. Besides protein degradation, lysosomes also function as a central hub for other organelles [132]. They continuously interact with endosomes, phagosomes, autophagosomes, mitochondria and PMs. Lysosome–mitochondria contacts were recently proposed to aid mitochondria fission and facilitate the transfer of lysosome-derived metabolites into the mitochondrial matrix to assist metabolic reactions in mitochondria [133,134]. Lysosomes also fuse with the PM to discharge their contents outside the cell [135]. These interconvertible membraneous compartments constitute the endo-lysosomal system, which is critical in maintaining cellular Cu homeostasis. Recent studies suggest their Cu storage and regulatory role to prevent cytotoxicity when the intracellular Cu supply is in excess [134,136,137]. When Cu exceeds the safe intracellular level in hepatocytes, ATP7B is exported from the Golgi to endo-lysosomal compartments, suggesting the involvement of lysosomes in mediating Cu efflux [136–141]. Studies in Cu^2+^ overloaded hepatocytes showed that Cu accumulated in the lysosomes and generated ROS, collectively causing a loss of lysosomal membrane integrity. Considering that the release of lysosomal proteases and phospholipases contribute to cytotoxicity, this collective evidence suggests lysosomes are likely to be the major site of endogenous cytotoxic ROS formation [142].

The cell surface abundance of CTR1 is tightly regulated through the endo-lysosomal system [143]. Upon Cu stimulation, CTR1 internalizes through endocytosis and rapidly enriches in early endosomes [41,144]. Once the normal Cu level is restored, CTR1 is sorted to the recycling endosomes and resupplied to the cell surface [41]. Recent studies by non-biased proteomic screening found that CTR1 also takes a retromer-dependent recycling route [145]. This finding links endosomes to the TGN for regulating intracellular Cu homeostasis and platinum-based drug uptake [141,145]. However, prolonged high Cu stress also leads to CTR1 degradation, presumably in the lysosome [144]. It is worth noting that CTR2, a highly conserved CTR1 homologue, is also located at the late endosome and lysosome. CTR2 mediates the formation of CTR1 ectodomain truncation and modulates CTR1 distribution to the cell surface, which prevents Cu accumulation in the endosomal compartment. The involvement of lysosomes in Cu homeostasis is not just for degrading CTR1. Studies on ATP7B showed that lysosomes could also serve as intermediate compartments for dispersing ATP7B from TGN under Cu stress [118,136,146]. Taken together, accumulating evidence supports the role of lysosomes in modulating Cu homeostasis [38,147].

Abnormalities in both endosomes and lysosomes, or dysregulation in their trafficking, have been associated with AD, PD and Lewy body dementia (reviews in [102,148,149]). The dysfunction of the endo-lysosomal system likely leads to the failure of clearance for amyloid proteins in the brain and the accumulation of toxic protein aggregates over time [149]. Concomitantly, Cu precipitation is often observed in patient brain lesions, and the association of Cu with amyloid proteins has been shown to accelerate senile plaque formation. Therefore, trafficking in the endo-lysosomal system has emerged as a common biological pathway affecting amyloid protein clearance and intracellular Cu balance in the neuronal system [5,102,150].

#### 3.1.4. Autophagosomes

Autophagosomes are another group of degradation compartments that continuously engulf organelle waste and deliver it to lysosomes for degradation. The dysfunctional autophagic flux (i.e. a measure of autophagic degradation activity) contributes to the deficient elimination of abnormal and toxic protein aggregates and is commonly seen in several major neurodegenerative disorders [134].

Several factors, including ER stress, oxidative stress and aging, affect autophagic flux. For example, Cu-induced oxidative stress can initiate the autophagy process in different tissues such as the kidney, liver and brain [151–153]. A recent study revealed that Cu-induced autophagy in MEFs is through the binding of Cu to autophagic kinases ULK1/2, which leads to an increase in autophagy flux in a dose-dependent manner [154]. Similar phenomena were also observed in dopaminergic cells, which show increase autophagic flux and protein ubiquitination under Cu stress [153].

Interestingly, autophagy impairment has been shown to impact intracellular Cu distributions and induce Cu toxicity [153]. It is known that autophagic activity decreases with age. Masaldan *et al.* identified Cu accumulation as a universal feature of senescent cells, whose enrichment is considered a hallmark of ageing. Elevated Cu in senescent MEFs was accompanied by elevated levels of Ctr1, diminished levels of Atp7a and enhanced antioxidant defense. They also found that rapamycin treatment, an mTOR inhibitor that activates autophagy in senescent cells, can prevent and reverse Cu accumulation, suggesting the protective role of autophagy in defending Cu-mediated damage [155]. These results suggest a close link between Cu homeostasis and the autophagic–lysosomal pathway [156,157]. In fact, several anti-cancer drugs have been developed based on this connection. For instance, Cu compound Casiopeina III-ia significantly inhibited the proliferation of glioma cells (i.e. tumour cells originated from glial cells) by simultaneously inducing autophagy and apoptosis [158]. However, the detailed mechanisms about how autophagosome counterbalance Cu dyshomeostasis are still unclear and need further investigation.

### 3.2. Regulators for Cu transporters trafficking between membrane compartments

Newborn Cu transporters CTR1 and ATP7A/B, like other integral membrane proteins in the cell, are synthesized and matured along the secretory pathway and further allocated to the PM or resided in the secretory pathway's endocytic branches, respectively, at a steady state. In response to Cu changes, Cu transporters travel in cells via vesicular networks, called endosomal networks, to change their cellular distribution (figure 4). Under Cu excess, cell surface CTR1 is reduced to minimize Cu uptake with concurrent ATP7A/B sequestering from the TGN to the PM to efflux the excessive Cu [125,159]. When Cu level resumes normal, CTR1 and ATP7A/B are sorted back to the PM and TGN via recycling and retrieval pathways, respectively. Endosomal networks are composed of dynamically interconnected trafficking compartments coordinated by multiple proteins responsible for phospholipid modification, cargo sorting, coat proteins assembly and motor protein tethering.

Numerous trafficking regulatory proteins have been identified to be closely associated with neuronal dysfunction when mutations occur [5,102]. Dysfunctional metal ion homeostasis may result in neurodegeneration and neuroinflammation, contributing to the development of several neurodegenerative diseases [101]. However, so far, only a limited number of trafficking regulators are identified to be responsible for the Cu-induced trafficking of Cu transporters, specifically CTR1 and ATP7A/B. Here, we review current knowledge about the known regulators involved in both Cu-transport trafficking and neurodegenerative diseases.

#### 3.2.1. Membrane trafficking regulators from internalization to degradation

Cells control the Cu influx by modulating the abundance and surface distribution of CTR1 [144]. CTR1 is distributed to the cell surface when cellular Cu is on-demand and internalized under Cu stress [42,122,160,161]. The internalized CTR1 can take the recycling endosome route when the Cu level is back to normal or the endo-lysosomal degradation route for permanent removal if cells encounter prolonged high Cu stress. CTR1 surface abundance seems to be cell-type specific [162,163]. Cells decrease the surface abundance of CTR1 under elevated extracellular Cu levels through clathrin-mediated endocytosis. It has been shown that blockages of clathrin-coated pit formation or pinch-off from the PM caused accumulation of CTR1 at the PM under a high Cu environment [41,144]. The initiation of clathrin-coated vesicles for CTR1 internalization is likely mediated by recruiting the adaptor protein complex AP-2 to the PM. It is known that the μ2 or β2 subunits of AP-2 recognize YXXØ or di-leucine motifs on the cytoplasmic domain of trafficking cargos [164,165]. It is also found that CTR1 contains a potential μ2-binding motif, YNSM, in its cytoplasmic loop. Mutations in this motif of CTR1 showed decreased CTR1 internalization, suggesting that the YNSM motif in CTR1 might be the site for μ2 binding [166]. However, attempts to detect direct interactions between CTR1 and adaptor subunits have not been successful, probably due to weak, transient interactions or a lack of other unknown interaction partners [166].

Upon internalization, CTR1 works closely with the conventional endocytic trafficking system. However, the molecular mechanisms directly regulating CTR1 trafficking are still less understood. Rab GTPases, the master regulators in orchestrating the identities and destinations of intracellular membrane vesicles, are likely to play an important role in CTR1 trafficking. The conversion of specific Rabs on trafficking vesicles determines the cargos’ fate throughout the secretory and endocytic pathways [167–171]. For example, Rab5 is enriched on the PM upon receptor activation to initiate the formation of early endosomes. Under Cu treatment, CTR1 is highly enriched in Rab5-positive endosomes [41]. Once internalized, Rab5-positive CTR1 vesicles are later either bound for the Rab7-dependent degradation pathway under high Cu dose or routed to the Rab11-dependent slow recycling pathway under transient Cu stimulation [41]. Despite knowledge about Rab GTPases, due to the lack of identified Cu-sensing regulators, it is still a mystery how the trafficking machinery responds to cellular Cu levels and delivers CTR1 accordingly.

ATP7A/B functions as Cu pumps responsible for cuproenzymes maturation in the secretory pathway and excessive cellular Cu efflux. Modulating the intracellular trafficking of ATP7A/B, instead of changing their expression levels, plays a prominent role in tuning ATP7A/B's functions [6]. Under the basal Cu condition, ATP7A/B mainly reside at the TGN and constitutively cycle between the TGN and PM [6]. The internalization of ATP7A from the PM can be mediated by clathrin/AP-2 complex-dependent and -independent pathways [115,172]. Under excessive Cu stress, most ATP7A is re-distributed to the PM and/or PM-adjacent vesicles [6,113,172]. The Cu-induced peripheral translocation of ATP7A requires reorganization of both actin and microtubule [9,172]. However, this translocation is independent of the integrity of the Golgi since Cu-induced ATP7A dispersing behaviour is still maintained in Golgi-fragmented cells [10,173]. Interestingly, although experimental results elucidating Cu-induced ATP7A/B degradation are scarce, ATP7A has been reported to be colocalized with Rab7, the landmark of the late endosome–lysosome system, under excessive Cu stress [174]. A recent study also showed that transient Cu exposure induces translocation of ATP7B to lysosomes followed by exocytosis [146], indicating that lysosomes have a distinct role in regulating Cu homeostasis. When the Cu level returns to normal, the dispersed ATP7A/B are internalized back to the endosomes and further retrieved back to the TGN. Some trafficking regulatory machinery for ATP7A/B has been nicely reviewed recently [9,175]. However, how the endocytic machinery senses Cu levels and modulates trafficking routes decision is still a mystery.

#### 3.2.2. Membrane trafficking regulators for recycling

Besides the internalized-degradation pathway, protein retrieval is another transient regulatory mechanism to re-distribute CTR1 and ATP7A/B when a normal Cu level is restored. Clifford *et al.* [41] demonstrated that CTR1 is sorted to the Rab11-dependent slow recycling pathway when cells are under low-dose Cu stimulation and relocate to the cell surface when the environmental Cu level returns to normal. Recently, unbiased systematic protein interactome studies further revealed that another retromer-mediated recycling pathway is involved in sorting CTR1 from degradation fate [141,145]. In retromer subunit-depleted cells, CTR1 fails to restore cell surface distribution after Cu wash-out. However, due to the lack of known sorting motif identified on CTR1, detailed mechanisms of how CTR1 is recognized by the retromer complex and sorted from endosomes need further investigations.

When cells restored normal Cu levels, the surface ATP7A/B, outbound through the secretory pathway, are internalized and further subjected to retrograde transport from endosomes to the TGN. The retromer and its associated protein complexes are the main players in mediating endosome-to-Golgi transport. The core of the retromer is the cargo-selective complex (CSC) VPS26A–VPS29–VPS35 heterotrimer, which works in concert with other cellular proteins to recycle CTR1 and ATP7A/B. The recycling is accomplished first through CSC recruitment to the endosomal membrane by sorting nexin 3 (SNX3) and Rab7. Once CSC binds to cargo, it further recruits membrane-deformation and tubulation proteins for the generation of the nascent cargo-loaded vesicles [176]. This process is coordinated with accessory proteins, including the Wiskott–Aldrich syndrome protein and SCAR homologue (WASH) and/or the COMMD/coiled-coil domain-containing (CCDC) 22/CCDC93 (CCC) complexes, to pack and transport cargo from endosomes to the Golgi. Although several retromer complexes were found to be involved in Cu-responsive retrieval of ATP7A/B, none of these retromer complexes and accessory proteins has been reported to bind to Cu, except COMMD1 [177,178].

COMMD1, previously called MURR1, is a membrane trafficking associate protein that specifically binds Cu in a 1 : 1 ratio with one methionine and two histidine residues [179]. COMMD1 directly interacts with ATP7A and ATP7B and is suggested as a regulator for Cu homeostasis [106,177,178,180]. COMMD1 is required for intracellular ATP7A/B trafficking through cooperation with the evolutionarily conserved WASH and retromer complex [181]. Deletion, mutation or depletion of COMMD1 or the CCC complex components abolishes Cu-dependent movement of ATP7A/B from endosomes, resulting in massive lysosomal Cu accumulation in livers and further leads to biliary excretion failure in dogs [182,183]. These observations indicate COMMD1 plays a critical role in the endosomal trafficking of ATP7A/B [181].

In addition to COMMD1, other general retrograde transport machinery regulators are also required to mediate ATP7A/B escape from degradation and proper relocation to the Golgi to restore intercellular Cu balance. These regulators include, but are not limited to, Rab22, clathrin coat protein, adaptor protein complexes AP-1/AP-2, retromer complex subunit VPS35, the WASH complex, sorting nexin, ADP-ribosylation factor 1 (ARF1) and the COG complex (see summary in table 2). These regulators play essential roles in cargo sorting and the formation of shuttling vesicles between endosomes and Golgi complex as well as Golgi tethering [115–118,130,131,141,181]. It is worth noting that none of these regulators has been reported to possess Cu-responsive motifs. It is still unclear how they recruit and dissociate ATP7A/B containing vesicles in response to cellular Cu changes. One possible explanation for the Cu-dependent trafficking is that the conformational changes on Cu transporters expose trafficking regulatory motifs upon Cu binding. Another possibility could be simply attributed to the involvement of un-identified Cu-sensing regulators.

**Table 2.** Intracellular regulators involved in Cu transporters trafficking.

copper transporter	membrane trafficking regulator	essential motif	key findings	implicated function of the regulator in the membrane trafficking process	ref.
*anterograde transport*					
CTR1	Rab11	un-identified	accumulated in Rab11-positive endosome upon Cu stimulation and translocated back to the PM when normal Cu level is restored	slow recycling endosome	[41]
*retrograde transport*					
CTR1	Rab5	un-identified	enriched at Rab5-positive vesicle at steady state	early endosome; essential for the assembly of clathrin-coated pits	[41]
	Rab9	un-identified	CTR1 lacking O-linked glycosylation is proteolytically cleaved in a Rab9-positive endosomal compartment	trafficking between lysosome and TGN	[121]
	AP-2 adaptor complex	YNSM^106^ (predicted)	Cu-induced CTR1 internalization via clathrin-dependent endocytosis. Inhibition of AP2-mediated clathrin coat assembling prevents the trafficking of hCtr1 from the PM	clathrin-coated assembly	[41,144,166]
	clathrin	YNSM^106^ (predicted); indirectly through μ2	knockout of clathrin light-chain abolished Cu-induced CTR1 endocytosis	clathrin-coated protein	[41]
	VPS35	Un-identified	failed to recycle back to the cell surface in VPS35 deficient cells	a component of retromer core subunits which mediates cargo retrieval from endo-lysosome	[141,145]
ATP7A	Rab22	un-identified	overexpression of dominant-negative Rab22a results in ATP7A punctate distribution	trafficking between Golgi apparatus and early endosome; Golgi retrieval	[115]
	clathrin	DKHSLL^1488^ di-leucine motif; indirectly through AP-2	plasma membrane accumulation in clathrin-depleted cells	clathrin-coated protein	[115]
	AP-2 adaptor complex	DKHSLL^1488^ di-leucine motif	plasma membrane accumulation in AP-2 depleted cells	clathrin-coated assembly	[115]
	AP-1 adaptor complex	un-identified; likely to be DKHSLL^1488^ di-leucine motif	punctate distribution in AP-1 depleted cells	associated with the sorting of cargo shuttling between endosomes and the TGN	[115]
	COMMD1	un-identified	ATP7A mislocated as puncta and failed to respond to Cu in COMMD1 depleted cells	a member of the CCC complex involved in retromer-mediated TGN retrieval from the endosome	[181]
	WASH complex & retromer complex	un-identified	Cu-induced ATP7A trafficking was impaired under WASH complex and retromer complex disruption	recruited to early endosome by CCC complex which involves in retromer-mediated TGN retrieval from the endosome	[181]
	SNX27	un-identified	ATP7A underwent lysosomal degradation in SNX27 and retromer-deficient HeLa cells	a component of SNX-BAR retromer which mediates endosome cargo sorting	[141]
	COG complex	un-identified	ATP7A interacts with COG subunits; cells lacking the COG complex shows increased surface ATP7A and decreased Cu content	Golgi complex tether	[130]
ATP7 (*Drosophila*)	COG complex	un-identified	ATP7 interacts with COG subunits; COG deficiency mitigates ATP7-mediated abnormal synaptic activity and mitochondria distribution	Golgi complex tether	[131]
ATP7B	AP-1 adaptor complex	DKWSLL^1455^ di-leucine motif	ATP7B lost somatodendritic distribution in neurons when either the di-leucine motif or AP-1 subunit were mutated	TGN retrieval	[116]
	Arf-1	DKWSLL^1455^ di-leucine motif	ATP7B has a strong binding with Arf-1 and AP-1 complex via di-leucine motif recognition	activator for AP-1 adaptor complex	[116,117]
	VPS35	^41^NVGY^44^ domain	ATP7B retrieval from the lysosome to TGN upon Cu-removal was impaired in VPS35 deficient cells	a component of retromer core subunits which mediates cargo retrieval from endo-lysosome	[118]
	COMMD1	un-identified motif at the amino-terminal tail	COMMD1 directly interact with ATP7B; knockdown of COMMD1 increased endogenous level of ATP7B	a member of the CCC complex involved in retromer-mediated TGN retrieval from the endosome	[106,107,178,184]

### 3.3. Dysfunction of Cu trafficking regulators links neural pathology

Patients with Cu transporter gene mutations showing neuropathological symptoms underline the involvement of Cu homeostasis in neurodegeneration. In addition to removing pathogenic protein aggregates, restoring proper Cu distribution is currently an important area for potential therapeutic interventions for neurodegeneration diseases [185]. Interestingly, the distribution of Cu transporters and the biosynthesis/clearance of pathogenic proteins are both subject to trafficking regulation. Mutations or dysfunctions of trafficking regulators have a broad-range impact on the intracellular distribution of overall membrane proteins, including Cu transporters and related Cu-required substrates, as well as organelle integrity and cellular behaviours and functions. Synergistically, perturbed trafficking could exacerbate Cu dysregulation and further devastate the symptoms by increasing the cytotoxicity of misfolded protein aggregates. Interactome studies have shown that multiple cytosolic trafficking-related molecules for Cu transporters are associated with diseases with neurological and/or neurodevelopmental phenotypes, emphasizing the collaboratory roles between membrane trafficking and Cu homeostasis in maintaining neural function [130,175]. Here, we take vacuolar protein sorting 35 (VPS35) and adaptor protein complex 1 (AP-1) as examples to discuss the interplay between Cu homeostasis and membrane trafficking regulation that potentially contributes to the pathogenesis of neurodegenerative diseases.

#### 3.3.1. VPS35/retromer in Parkinson's disease

Mutation of the VPS35 gene, encoded the core subunit of retromer, has emerged as a cause of late-onset familial PD [186–188] (summarized in review [189]). The effect of mutated VPS35, specifically the D620N variant, is attributed to the disruption in the formation of retromer transport carriers [190]. Such perturbations cause abnormal PM retrieval and endo-lysosomal trafficking after Cu depletion, which prevent CTR1 and ATP7B from trafficking back to the PM and TGN in non-neuron systems, respectively [118,145]. Although current results were obtained from non-neuron cells, it is reasonable to expect that the D620N mutant may cause Cu deficiency in the neuronal system. This perturbed Cu supply is reminiscent of Parkinson-like symptoms in WD and could explain the widespread cerebral Cu deficiency in PD dementia [191]. Further investigation using an appropriate neuronal model will provide valuable insight into these observations.

Paralleled with synaptic morphology, transmission and plasticity alterations, mitochondria fragmentation is a phenotype commonly observed in neurodegenerative diseases. From a membrane trafficking perspective, such mitochondria fragmentations can occur through abnormal mitofusin-2 (MFN2)-mediated fusion or dynamin-like protein 1 (DLP1)-mediated fission processes. Tang and colleagues showed that mutant VPS35 dysregulates the trafficking and minimizes the degradation of the E3 ubiquitin ligase MUL1, thus promoting MUL1-mediated MFN2 degradation and decreased mitochondrial fusion activity [192]. Similarly, mutant VPS35 also causes mitochondrial dysfunction by recycling DLP1 complexes, thus increases mitochondrial fission activity [193]. In both cases, mutant VPS35 causes mitochondrial fragmentation and dopamine neuron loss. On the other hand, dysregulated supply of Cu also devastates the destruction of mitochondria in MD and WD models [175,194–196]. Mitochondria from Atp7b^–/–^ rat liver shows progressive ultrastructure changes as Cu accumulates and eventually fragmented. [195] Such fragmentation is likely due to Cu overload stimulating hydroxyl radicals production that triggers free-radical damage of the abundant lipoprotein, cardiolipin [197].

Although both mutant VSP35 and Cu accumulation cause mitochondria fragmentation, the direct connection between the two likely happen in the dopamine signalling. Dopamine plays a key role in regulating various brain physiological functions by binding to its receptors for surface recycling and signalling. Studies in hippocampus neurons have demonstrated that axonal trafficking of mitochondria could be manipulated by dopamine receptor D2 (DRD2) agonists [198]. Cu is required for the activity of dopamine biosynthesis enzymes, including tyrosine hydroxylase and DBH. Cu deficiency leads to a shortage of dopamine supply and likely results in abnormal mitochondria trafficking. Interestingly, dopamine receptor D1 (DRD1), another subtype of dopamine receptor in hippocampal neurons that has the opposite effect to DRD2 on axonal mitochondrial trafficking, is another cargo of VPS35 and the associated retromer complex [198]. VPS35/retromer complex regulates DRD1 plasma membrane recycling and the downstream cAMP-response element-binding protein (CREB) and extracellular regulated protein kinase (ERK) signalling [145,199]. These lines of evidence suggest that the impact of dysregulated Cu in dopaminergic neurons can be either mediated by dopamine biosynthesis via impaired cuproenzymes activity or by affecting dopamine signalling pathways.

Toxic, misfolded α-synuclein aggregates in Lewy bodies are another pathological hallmark of PD, which can originate from elevated synuclein protein expression/aggregation or failure of cellular protein degradation systems. α-Synuclein possesses multiple Cu-binding sites, and the presence of Cu initiates oligomerization of α-synuclein and increases α-synuclein toxicities [200–204]. The overall Cu content does not vary between healthy and diseased brains. Reduced Cu and CTR1 expression in the cerebrum and increased Cu in the CSF are key features in PD, indicating the misdistribution of cellular Cu, rather than the total Cu content in the brain, is the essential factor for PD dementia [191]. Misdistribution of cellular Cu potentially could originate from abnormal cathepsin endo-lysosomal proteases activity, which controls Cu accumulation via cleavage of the Ctr1 metal-binding ectodomain [205]. Interestingly, the Vps35 D620N mutation has also been linked to disrupted trafficking of cathepsin D, a protease important for the degradation of α-synuclein, suggesting potential pathways of how Vps35 may affect Cu homeostasis and synergistically contribute to α-synuclein pathogenesis [206].

The lysosomal system is another major pathway for α-synuclein degradation and is considered a hub for maintaining Cu homeostasis [136,207,208]. Cu is significantly associated with lysosomes in primary cortical neurons. [209] The uptake and storage of Cu into lysosomes can be regulated by CTR2, a CTR1 homologue [38,147]. Under Cu overload, ATP7B enables lysosomes to undergo exocytosis for Cu clearance through the interaction with the p62 subunit of dynactin that allows lysosome translocation toward the canalicular pole of hepatocytes [136,137]. On the other hand, lysosomal vesicular sorting and trafficking can be modulated by VPS35 (D620N) mutation through enhancing the leucine-rich repeat kinase 2 (LRRK2)-mediated Rab protein phosphorylation [210–213]. Considering that LRRK2 regulates lysosomal protein trafficking and morphology [214,215] and VPS35 also cooperates with LRRK2 to regulate synaptic vesicle recycling and dopaminergic synaptic release [216], it is likely that mutation in VPS35 may lead to abnormal lysosomal activity and consequently inefficient α-synuclein degradation [217]. This collective evidence further supports the systematic role of VPS35/retromer and Cu homeostasis in α-synuclein expression, accumulation and aggregation, which all contribute to the pathogenesis of PD [201].

#### 3.3.2. AP-1 complex in neuropathological symptoms

MEDNIK (acronym for mental retardation, enteropathy, deafness, peripheral neuropathy, ichthyosis and keratoderma) and MEDNIK-like syndromes are rare autosomal recessive neurocutaneous diseases that show some similar clinical and biochemical phenotypes of both MD and WD. For example, MEDNIK patients show MD-like reduced plasma Cu and ceruloplasmin level and WD-like liver Cu accumulation and increased urinary Cu excretion. MEDNIK-like patients have low plasma Cu and ceruloplasmin phenotypes but lack hepatic Cu toxicity evidence [218]. Regarding neuronal-related phenotypes, MEDNIK, MEDNIK-like, MD and WD patients all show cerebral atrophy. Still, the symptoms in MEDNIK and MEDNIK-like patients are typically milder than the MD and WD patients [175,185,207,218].

MEDNIK and MEDNIK-like syndrome are associated with mutations in the adaptor protein-1 S1 (*AP1S1*) and B1 (*AP1B1*) gene, respectively. AP1S1 and AP1B1 encode for the small subunit σ1A and large β subunit of the AP-1 complex, which interact with clathrin and incorporate their cargos into clathrin-coated vesicles. The AP-1 complex is involved in sorting transmembrane proteins en route for the TGN or endosomes, somatodendritic sorting in neurons [219,220] and basolateral sorting in the epithelium [221–223]. Given the similar Cu imbalance phenotypes observed in MEDNIK, MD and WD, the Cu metabolism defects are reasoned to be the abnormal retrieval of Cu-ATPases due to mutations of the AP-1 complex. Research in rat hippocampal neurons has shown that the di-leucine-based signal motif of ATP7B strongly interacts with the σ1 subunit of AP-1, contributing to the somatodendritic polarize sorting of ATP7B [116]. Given that ATP7A and ATP7B are structurally similar, AP-1 mutant may lead to aberrant trafficking and impair both ATP7A and ATP7B functions, resulting in Cu-related characteristics of MD and WD. Indeed, fibroblasts from MEDNIK patients display abnormal subcellular distribution of ATP7A, which accumulates at the cell periphery instead of concentrating in the Golgi region [224]. The MD-like reduced plasma Cu and ceruloplasmin level and WD-like liver Cu accumulation phenotypes seen in MEDNIK patients can be explained by the perturbed polarized distribution of ATP7A and ATP7B in enterocyte and hepatocytes.

Although adapter protein complexes with mutations on various subunits are associated with neuropathy (summarized in Guardia *et al.* [225]), only AP1S1 and AP1B1 mutation showed Cu metabolic perturbation symptoms. Due to the complexity of intracellular trafficking machinery, the exact mechanism of AP-1 mediated trafficking of Cu transporters, leading to preferential Cu metabolic phenotypes, is still unclear. It is suspected that additional factors cooperating with the AP-1 complex exacerbate the impact of misregulated ATP7A. ARF1, the AP-1 complex activator, is one of the potential candidates due to its involvement in both retrograde transport to TGN and is required for maintaining Golgi ribbon integrity and biogenesis of ATP7A [117,173,226]. Interfering ARF1 function by using RNAi-mediated ARF1 depletion or ARF1 dominant-negative mutant overexpression caused the dispersion of the TGN and ATP7A as well as dissociation of AP-1 complex from the membrane [173]. Nevertheless, these aligned pieces of evidence support the importance of the AP-1 complex in mediating the TGN-bound trafficking of ATPases, specifically ATP7A, in maintaining Cu balance.

In addition, the AP-1 dysfunction induced abnormal ATP7A/B distribution may contribute to neuronal-specific phenotypes by disrupting systematic Cu homeostasis and neurotransmitter activation. It is known that ATP7A accumulates at the cell periphery in MEDNIK patients, and the concentration of ATP7A in the Golgi region likely to be lower. The low ATP7A in the Golgi region provides a possible explanation for its connection to neuronal-related phenotypes through disrupted interactions between ATP7 and other Golgi regulatory machinery such as the COG complex. Evidenced by using the *Drosophila* model, Hartwig *et al.* demonstrated that interactions between ATP7 paralogs and COG complex, a Golgi apparatus vesicular tether, are essential to maintain Cu homeostasis in neurons. Disruption of ATP7–COG complex interaction affects COG-mediated TGN proteins recycling in motor neurons, which is similar to manipulating the expression level of ATP7 paralogs, leading to the perturbation of Cu homeostasis, decreased synaptic mitochondria content and altered synapse plasticity [131].

Another important protein that may contribute to the neurological symptoms of MEDNIK and also encompass Cu homeostasis is PAM. AP-1 has been shown to regulate the endocytic trafficking of PAM in neuroendocrine cells. Co-immunoprecipitation of the AP-1 and a cytosolic-domain truncated PAM protein suggest that luminal domains of PAM could be involved in the interaction. The proteins that contribute to this interaction have not been identified. However, it has been shown that luminal fragments of ATP7A interact with PAM while delivering Cu, suggesting that ATP7A is a possible intermediate in this AP-1 and PAM interaction [227,228]. Indeed, reduced AP-1 level causes PAM's activity reduction to be more sensitive to Cu restriction. Since PAM does not bind Cu tightly, it is also suggested that the cell surface retention of PAM caused by AP-1 dysfunction leads to their Cu loss and thus diminished amidation function [229]. Impairing AP-1 function in neuroendocrine cells, which leads to the sensitivity of peptide amidation to Cu availability, could restrict peptidergic signalling and contribute to the complex phenotype.

## 4. Conclusion remarks and perspectives

This review summarized current knowledge of the general trafficking itineraries for Cu transporters under different Cu conditions and highlighted several critical membrane trafficking regulators in maintaining Cu homeostasis. Yet, a detailed molecular understanding of the trafficking machinery of Cu transporters is still beyond reach due to multiple unsolved questions. One of the unsolved questions is how trafficking machinery senses Cu levels and modulates the distribution of Cu transporters through membrane trafficking. Since Cu is the ligand for Cu transporters, one possible hypothesis is that Cu transporters may change their protein structure or oligomerization upon Cu binding to regulate trafficking routes. Current knowledge learned from surface receptors, such as GPCRs, showed that ligand binding could induce receptor dimerization coupled with conformational changes, which exposes their regulatory motifs to recruit trafficking machinery to the receptor [230–232]. Manipulation of the oligomeric status of membrane proteins could also modulate the turnover of the trafficking regulators and the maturation progress of vesicles, which eventually affected the overall cell behaviours [231,233,234]. So far, most of the structural and conformational studies on Cu receptors have relied heavily on purified proteins and *in vitro* biochemical assays, which may not faithfully reveal the dynamical behaviour of membrane proteins in cells.

Another intriguing question is whether there is Cu-specific regulatory machinery for Cu-induced membrane trafficking. Current findings unanimously show that the same trafficking machinery is shared in distributing membrane proteins, including Cu transporters, in the cells. However, only Cu transporters are sensitive to Cu levels and subject to Cu-induced redistribution [41]. It is tempting to speculate that some regulators might directly associate with Cu and serve as a Cu-specific trafficking regulator. COMMD1, by far, is the only identified membrane trafficking regulator with Cu-binding capability that directly mediates cellular Cu homeostasis [106,179,180]. Recent studies in tissue-specific knockout mice suggested that, within the COMMD family, Commd6 and Commd9 might also play a similar role as Commd1, since mice with liver-specific deficiency on Commd1, Commd6 or Commd9 shared the same Cu accumulation phenotype in the liver [235]. Further, COMMD1 has been reported to regulate the trafficking of ATP7A and ATP7B, whose functions and distributions are similar but not identical [86]. It suggests that there might be other unidentified regulators responsible for their respective membrane trafficking.

Besides these outstanding questions, several challenges remain to be resolved before understanding the interplaying mechanisms of Cu homeostasis and membrane trafficking in human neurons. Current molecular understanding of Cu transporters trafficking is mainly originated from non-neuronal cells. This could be a concern since Cu homeostasis and the trafficking of Atp7a show drastic differences between mice intestine and liver cells [235]. This observation suggests potential differences in the trafficking of neurons and non-neuronal cells that were primarily neglected in the past. It is critical to revisit Cu transporters trafficking in proper neuronal models to build a solid foundation for the field. Recent progress of human embryonic stem cells (hESCs)/inducible pluripotent stem cell (iPSC)-derived neurons have shown that they can faithfully recapitulate an individual's idiosyncratic neural development. Generations of knock-in stem cell lines expressing fluorophore-tagged Cu-binding proteins could provide an ideal platform for studying the causality of the mutations of diseases [236,237]. Furthermore, the recent advance in super-resolution microscopy enables researchers to approach biophysical problems like protein kinetics and oligomeric states from a single-molecule perspective. For example, using single-molecule tracking, Chen *et al.* [238] discovered the unbinding kinetics of MerR-family metalloregulators from operator sites could be modulated by their cellular concentration and chromosome organization. In combination with single-molecule diffusion analysis [238], they also identified that that CusC_3_B_6_A_3_ complexes, a tripartite RND-family Cu(I) and Ag(I) efflux pump, are dynamic structures and shift toward the assembled form in response to metal stress [239]. We recently developed a new method to quantify oligomeric states of membrane proteins using super-resolution localization [240,241] and can help understand cellular tasks mediated by the transitions between different oligomeric states. These new results show that single-molecule localization microscopy (SMLM) can follow protein complex formation, interconversion and dissociation in real-time. It also circumvents the general challenge of studying protein behaviours *in vitro*, where protein complex reconstitution is technically demanding and mimicking the cellular environment is almost impossible. Most importantly, SMLM shifts quantifications of specific protein behaviours from *in vitro* to physiologically relevant human cells for biophysical research.

Being the most polarized, morphologically diverse and not-dividing cell type, neurons are extremely active in membrane trafficking to maintain proper functions. Perturbation of trafficking regulation related to Cu transporters' cellular distribution and dysfunctional endocytic machinery are often observed in neurodegenerative neurons [12,242–246]. Hampered by neurons’ compact and complex morphology, the studies of Cu transporters' organization and response in live neurons have not been achieved. We expect the aforementioned technical challenges to be resolved with the thriving super-resolution imaging techniques and neuronal differentiation from patient-derived stem cells. Information from these studies will shed light on our understanding of Cu transporters’ physiological configurations, signalling and behaviour dynamics in maintaining neuronal Cu balance.

## Data accessibility

This article has no additional data.
